# Comparable Pregnancy Loss and Neonatal Birthweights in Frozen Embryo Transfer Cycles Using Vitrified Embryos from Progestin-Primed Ovarian Stimulation and GnRH Analogue Protocols: A Retrospective Cohort Study

**DOI:** 10.3390/jcm11206151

**Published:** 2022-10-19

**Authors:** Weiran Chai, Maokun Liao, Guang’en Feng, Mengjie Wei, Wentao Shi, Yun Wang, Qiuju Chen

**Affiliations:** 1 Department of Assisted Reproduction, Shanghai Ninth People’s Hospital Affiliated to Shanghai Jiaotong University School of Medicine, Shanghai 200011, China; 2 Clinical Research Unit, Shanghai Ninth People’s Hospital Affiliated to Shanghai Jiaotong University School of Medicine, Shanghai 200011, China

**Keywords:** assisted reproductive techniques, pregnancy loss, live birth, progestin-primed ovarian stimulation, GnRH analogue

## Abstract

Background: The potential correlation between progestin-primed ovarian stimulation (PPOS) and the risk of compromised embryo competence still lacks sound evidence. Methods: A large retrospective cohort study was used to compare the incidence of pregnancy loss and neonatal birthweights in frozen embryo transfer (FET) cycles using embryos from PPOS and GnRH analogue protocols. Propensity matched scores were used to balance the baseline confounders. Results: A total of 5744 matched cycles with positive hCG test were included to compare the pregnancy outcomes. The incidence of pregnancy loss was similar between PPOS and GnRH analogue groups (19.2% vs. 18.4%, RR 1.02 (0.97, 1.06), *p* > 0.05). The neonatal birthweights were comparable between two groups, respectively, for singleton births (3337.0 ± 494.4 g vs. 3346.0 ± 515.5 g) and in twin births (2496.8 ± 429.2 g vs. 2533.2 ± 424.2 g) (*p* > 0.05). Conclusions: The similar incidence of pregnancy loss and neonatal birthweights in FET cycles using embryos from PPOS provided us with a more complete picture about the safety of PPOS.

## 1. Introduction

Controlling premature ovulation is a key challenge in the field of assisted reproductive techniques. Gonadotropin-releasing hormone (GnRH) analogues (agonist and antagonist) are widely used to suppress pituitary activity and prevent the premature surge of luteinizing hormone (LH); their efficacy and safety have been confirmed during forty years of practice [1,2]. Recently more flexible protocols were proposed with the aid of vitrification, for example, progestin was extensively used for the prevention of premature ovulation as a supplement to GnRH analogue [3,4]. This progestin-primed ovarian stimulation (PPOS) has become a feasible protocol in patients undergoing ovarian stimulation for freeze-all IVF [3].

However, the potential of PPOS in clinical practice remains to be determined; specifically, it is still unknown whether the ovarian stimulation with the adjunctive use of progestin impacts the oocyte quality and therefore embryo competence, in terms of epidemiological investigation. Previous data from clinical trials showed PPOS had similar oocyte yields and pregnancy outcomes comparing the short protocol in the freeze-all context [4,5], GnRH antagonist protocol [6,7] and GnRH agonist ultra-long protocol [8]. Notably, some retrospective studies recently suggested that progesterone elevation may impair oocyte and embryo quality [9,10,11]. Another prospective clinical trial indicated that the early miscarriage of PPOS was comparable with GnRH agonist or antagonist protocols, although the statistical power was limited by the small sample size [3]. Moreover, few reports focused on the monitoring of the whole pregnancy process. There is still a lack of data on pregnancy loss after confirmed a serum positive hCG test. The incidence of pregnancy loss after confirmed serum hCG positive tests would provide valuable evidence for evaluating the embryo competences in IVF/ICSI treatments.

To investigate the potential correlation between PPOS and the risk of compromised embryo competence, we performed a large retrospective trial to compare the incidence of pregnancy loss and neonatal birthweights in frozen embryo transfer (FET) cycles using embryos from PPOS and conventional GnRH analogue protocols. Propensity matched scores were used to balance the baseline confounders [12]. The pregnancy loss in the PPOS group was further explored, using stratification by maternal age and oocyte yields.

## 2. Materials and Methods

### 2.1. Study Design

From 2011 onwards, our clinic has promoted freeze-all IVF cycles for the general infertile population and a large number of PPOS cycles were completed since 2014, which provided a good chance to investigate their effectiveness and safety. We did a retrospective cohort trial to evaluate the prevalence of pregnancy losses and live birth outcomes in FET cycles using vitrified embryos that originated from PPOS and conventional GnRH analogue protocols during 2014–2017. The use of data in this study was authorized under the IRB of Shanghai Ninth People’s Hospital. No consent forms were needed for the retrospective study; we ensured that the patients’ information was anonymous and only used for research.

### 2.2. Study Population and Data

To widen the generality of our findings, a wide enrollment criterion was applied; eligible cases were defined as women with maternal age no more than 43 years, with normal ovarian reserve (including total AFC ≥ 5 and basal FSH < 10IU/mL) and completed freeze-all cycles. Because the pregnancy loss and neonatal outcomes were the outcomes of interest in this study, we only included cycles with confirmed serum hCG positive test at 14 days after FET. The FET cycles with unknown pregnancy outcome were excluded. The database included all the parameters of demographic data, embryo characteristics, pregnancy outcome and, if pertinent, obstetric outcomes. No imputation for missing data was planned. The study population was divided into two groups based on the embryo origins from the ovarian stimulation protocols (PPOS group and conventional protocols using GnRH analogue group).

For the PPOS group, gonadotropin 150–225 IU/d and oral progestins were administered from menstrual cycle day 3, and the daily dose and types of progestin included MPA 10 mg, dehyprogesterne 20 mg or micronized progesterone 0.2 g, respectively. Previous clinical trials showed no difference of pregnancy outcomes in the comparison of different types of progestins [4,13,14]. When more than 3 dominant follicles reached 18 mm, GnRH agonist 0.1 mg and a low dose of hCG (1000 IU–2000 IU) were used as triggers and oocyte retrieval was arranged 36 h later. The control group included the conventional protocols using GnRH analogue (GnRH agonist long protocol, short protocol and GnRH antagonist protocol). The cases did not include fresh embryo transfer.

All good-quality embryos were vitrified and cryopreserved on day 3. Non-good- quality embryos were allowed extended culture into blastocysts and only good-morphology blastocysts were cryopreserved. After one menstrual cycle at least, the endometrium was prepared by the natural cycle, mild stimulation or hormonal replacement therapy (HRT), and embryos were transferred in a more natural-like cycle. Up to two embryos were transferred in one cycle. Details about ovarian stimulation and endometrium preparation were described in the reports of previous trials [4,13].

The pregnancy cases completed three ultrasound examination visits at six, eight and ten weeks of gestation age in our clinic, then were scheduled to attend the antenatal care in local maternity hospitals. Our trained nurses completed telephone surveys during each trimester of pregnancy and up to 1 week after delivery. Standardized questionnaires were used to gather information including a wide range of pregnancy complications, gestational ages, mode of delivery, birth date and locality, birthweights, newborn gender as well as neonatal death. In cases of failed attempts to contact the couples, information was collected through the local agencies of the family planning service.

### 2.3. Outcomes

The primary outcomes of interest were the incidence of pregnancy loss and the neonatal birthweight. Pregnancy loss was defined as the demise of pregnancy before 24 weeks of gestation age, including the biochemical pregnancy, ectopic pregnancy, early miscarriage and late miscarriage. The biochemical pregnancy was defined as pregnancy demised based on decreasing serum or urine beta-hCG levels, without an ultrasound evaluation. Early miscarriage was defined as intrauterine pregnancy loss before 12 weeks’ gestation and late miscarriage was defined as pregnancy loss after 12 weeks’ gestation. Live birth was defined as the delivery of a viable infant after the 24th gestational week. Gestational age (GA) in FET cycles was calculated from the day of embryo transfer, which was defined as day 17 for cleavage stage embryo transfer and day 20 for the blastocyst transfer. Preterm birth was defined as delivery before 37 completed gestational weeks. The pregnancy complications (including gestational diabetes, hypertension, pre-eclampsia and intrahepatic cholestasis syndrome), live birth defects, newborn gender and gestational age at birth were also compared between the two groups.

### 2.4. Statistical Analysis

The baseline characteristics and neonatal outcomes were compared with Student *t* test for continuous data, and *χ*^2^ or Fisher’s exact tests for categorical data, as appropriate. The propensity scores were calculated using logistic regression based on patient and cycle characteristics including maternal age, paternal age, BMI, duration of infertility, IVF indications, baseline FSH values, gravidity, number of previous IVF failures, antral follicle counts (AFC), oocyte yields, endometrium thickness and the number of transferred embryos. Matches without replacement were performed using propensity scores through the nearest neighbor random matching algorithm [12]. The matched ratio for GnRH analogue versus PPOS was 1:2. The prevalence of pregnancy loss, preterm and term birth were calculated in the cases of matched groups. Singleton and twin newborns were compared separately.

Sensitivity analysis was performed to compare the incidence of pregnancy loss and neonatal birthweight using the original data. The pregnancy loss and perinatal outcomes are listed in Supplemental Table S1. The multivariable logistic regression analysis was used in the original data to compare the pregnancy loss outcomes. To exclude the negative influence of pregnancy complications on fetal growth, the perinatal outcomes in matched cases without pregnancy complications were also compared. Statistical analysis was done using SPSS 24.0 and SAS Enterprise Guide version 7.1. A *p*-value < 0.05 was considered to indicate statistical significance.

## 3. Results

### 3.1. Study Population and Baseline Characteristics

**Figure 1:**
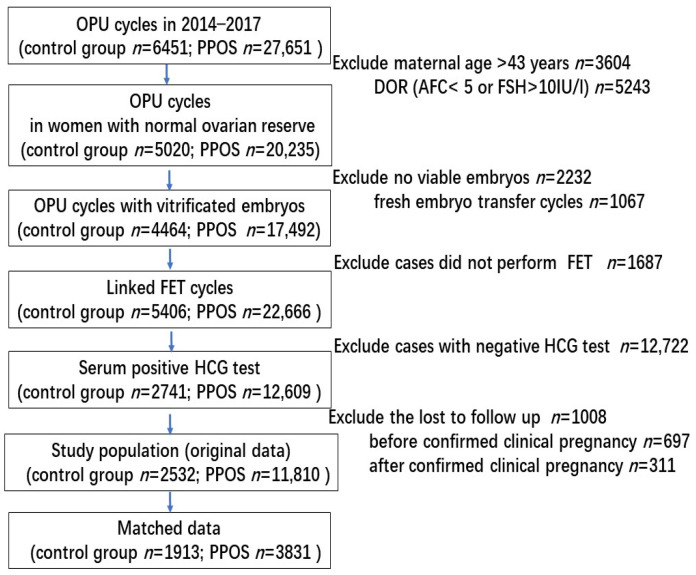
Screening and flow of participants through study.

A total of 11,810 positive hCG cycles with embryos originating from PPOS and 2532 cycles originating from conventional GnRH analogue protocols were enrolled from January 2014 through December 2017. The screening procedure is shown in Figure 1.

The demographic and clinical characteristics of the original population are shown in Table S1. The patients of PPOS groups in the original data tended to be younger, with lower BMI, longer infertility duration and less IVF failures compared with the control group. Gravidity, parity, prior spontaneous and induced abortions were comparable between the two groups. Cycle-specific factors showed no difference in terms of fertilization methods, endometrium thickness and the number of transferred embryos, but slightly higher oocyte yields in the PPOS group (*p* < 0.05).

In combination with the possible associated factors identified in a preliminary literature search, we subsequently performed propensity score matching to control the balance. The propensity scores were calculated using logistic regression based on patient and cycle characteristics as above described. AMH was not considered for inclusion due to a large percentage of data missing (85% missing). We selected a 1:2 match between control and PPOS groups by propensity score matching and all the confounding factors were balanced (Table 1). The density of the propensity score in original and matched data is shown in Supplemental Figure S1.

### 3.2. Incidence of Pregnancy Loss

The pregnancy outcomes of the matched data are shown in Table 2. The total rate of pregnancy loss was 19.2% in PPOS and 18.4% in GnRH analogue groups, with no significant difference (RR1.02 (0.97, 1.06), *p* > 0.05). Using the unmatched original data, the total rate of pregnancy loss was 18.9% in the PPOS and 19.2% in control groups. After adjustment for factors including the maternal age, paternal age, BMI, infertility duration, IVF failures and the oocyte yields, the comparison of PPOS versus GnRH analogue group showed no significant difference (aOR 0.97 (0.87, 1.08), *p* > 0.05) (Table S2).

The details of pregnancy loss are listed in Table 2. The loss rates due to biochemical pregnancy (3.9% vs. 2.3%), early miscarriage (8.7% vs. 11.9%), late miscarriage (3.5% vs. 2.4%), ectopic pregnancy (3.0% vs. 1.8%) were slightly different between the PPOS and GnRH analogue groups (*p* < 0.05).

### 3.3. Neonatal Birthweights in Singleton and Twin Births

The prevalence of live births was 80.8% in the PPOS group and 81.6% in the GnRH analogue group, using the matched data, and did not reach significant difference (*p* > 0.05) (Table 2).

In this data, the neonatal birthweight was missing for 9.0% and birth gender was missing for 15%. Single births occurred at an average of 38.5 weeks and nearly one half of twin births were delivered before 37 weeks. The total neonatal birthweights were comparable between two interventions, respectively for singleton birth (3346.0 ± 515.5 g in the control group and 3337.0 ± 494.4 g in PPOS group, *p* > 0.05) and twin births (2533.2 ± 424.2 g in the control group and 2496.8 ± 429.2 g in PPOS group, *p* > 0.05) (Table 2). The proportion of preterm delivery, newborn gender, incidences of low birthweight and high birthweights showed no significant difference between the two groups (*p* > 0.05). The incidence of congenital anomalies was 2.6% and 1.9%, respectively, for the PPOS and GnRH analogue groups (*p* > 0.05).

### 3.4. The Incidence of Pregnancy Loss Stratified by Maternal Age and Oocyte Yields

**Figure 2:**
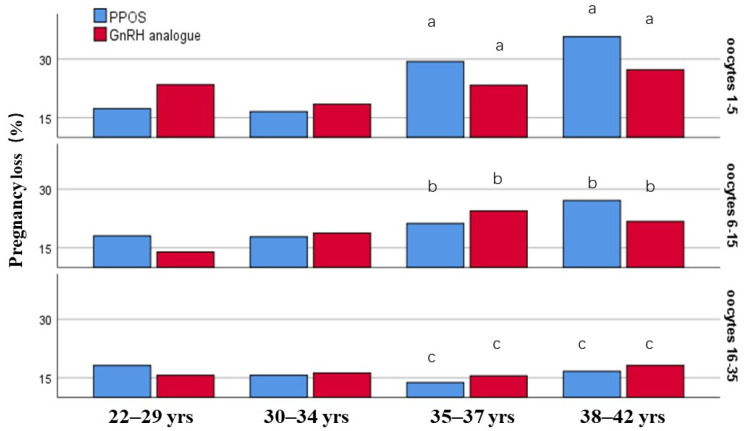
The incidence of pregnancy loss was stratified by maternal age and the retrieved oocytes. Different letter means significant difference among the subgroups of different oocyte yields (*p* < 0.05).

The pregnancy loss may be associated with maternal age and the ovarian reserve, so the incidence of pregnancy loss was compared using stratification by maternal age and the oocyte yields. In this study, the comparison of the pregnancy loss rate between PPOS and GnRH analogue group did not show significant differences in the subgroups for maternal age and oocyte yields (*p* > 0.05) (Figure 2).

To be noted, the incidence of pregnancy loss increased with increasing maternal age in the subgroups of retrieved oocytes 1–5 and 6–15, while it did not increase with age in the groups of oocytes 16–35. Especially at advanced age ( ≥35 years), the incidence of pregnancy loss decreased with the increasing oocyte yields, which indicated that the pregnancy loss was negatively associated with the number of retrieved oocytes in women more than 35 years (Figure 2).

### 3.5. Sensitivity Analysis

The multiple logistic regression for original data was used as sensitivity analysis and the factors such as the maternal age, paternal age, BMI, infertile duration, IVF failures and the oocyte yields were adjusted in the logistic regression model. No significant difference was found in the pregnancy loss rate and neonatal birthweights between PPOS and GnRH analogue groups, in line with the results of matched data (Table S2). In the PPOS group, the incidence of high birthweight was slightly lower (14.5% vs. 16.8%) and the incidence of birth defects was higher (2.6% vs. 1.7%), which may be associated with the higher prevalence of twin pregnancies in PPOS group.

Given that pregnancy complications may affect intrauterine fetal growth, we performed an analysis excluding this factor in the matched data (*n* = 5453). As seen in Table S3, the results of birthweights in cases without pregnancy complications were similar between the two groups (*p* > 0.05).

In addition, the distribution of endometrial preparation regimen and embryo stage showed a slight difference between the two treatments in the matched data. The three regimens for FET were widely accepted and no evidence supported the use of one regimen over another [15]. We performed subgroup analysis to investigate the influence of embryo stages for pregnancy outcomes. The results showed the same trend between the two treatments whether using cleavage embryos or blastocysts (Table S4).

## 4. Discussion

In the large cohort study, we observed that PPOS did not increase the risk of pregnancy loss compared with GnRH analogue protocols in the freeze-all context. We also reported the neonatal birthweights stratified by fetalis and found no obvious differences of live birth outcomes between the PPOS and the control group. These findings confirmed the safety of PPOS in terms of pregnancy outcomes.

Although attractive and promising, the available evidence on PPOS is limited; its exact role and reproductive outcomes in freeze-all cycles are still in doubt, especially as regards the quality of oocytes and embryos [3,16]. Follicles express two types of progesterone receptors, progesterone receptor (PGR) and PGR membrane component 1 (PGRMC1); while PGR shows transient expression on granulosa cells of graafian follicles, PGRMC1 expresses in granulosa cells of developing follicles, so progesterone may control the growth of developing follicles through PGRMC1 [17]. Adding progesterone to in vitro culture medium significantly inhibited oocyte meiotic resumption in a dose-dependent manner, thus leading to an increase of germinal vesicle arrest and reduction in oocyte maturation [18]. Previous evidence in clinical studies showed that continuous administration of oral progesterone did not harm clinically recognized indicators of oocyte/embryo competence such as the oocyte yield, embryo implantation rate or clinical pregnancy rate in PPOS compared with the GnRHa short protocol [4,5,6,7,8,13,14]. However, these trials were not long or large enough to assess the efficacy of the whole pregnancy.

Our study with the precise date of pregnancy after FET and the longitudinal observation for the whole gestation age provided an intact overview for the status of pregnancy loss in a large sample of PPOS cycles. Our study was pragmatic, with the aim of describing a real-world environment. We did not limit the progesterone type or the clinicians’ preference in selecting the regimen of ovarian stimulation, in order to draw a more robust conclusion for generalization. In our clinic, the new established protocols of PPOS gained recognition since 2015 and became dominant in a short time [4,13,14,19,20]; the GnRH analogue protocols were a relatively small proportion of ovarian stimulation (17%). After propensity score matching, the covariates were mostly in good balance; the overall frequency of pregnancy loss among positive hCG women was 19.2% in the PPOS group and 18.4% in conventional protocols, comparable with previous reports of GnRH analogue cycles [1]. The pregnancy loss rate in the matched data were also similar with the findings of original data using multivariable logistic regression. These data showed PPOS did not increase the incidence of pregnancy loss, indicating that the current progestin administration did not show a detrimental effect on oocyte potential.

Live birthweight is a good indicator of embryo quality in IVF/ICSI treatments [21]. The proportion of twin pregnancies was slightly higher in the PPOS group and the average birthweights were comparable after adjusted the fetus numbers. The incidence of congenital anomalies did not increase in the PPOS group, which was in line with previous reports [19,20,22]. These results of live births provided further evidence of embryo competence in PPOS treatment.

Maternal age and ovarian reserve were two critical factors for pregnancy loss [23]. Using stratified analysis by maternal age and the oocyte yields (a surrogate marker of ovarian reserve), the pregnancy outcomes did not show obvious differences between PPOS and GnRH analogue, but in the older women (≥35 years), the ovarian reserve clearly affected the incidence of pregnancy loss. Thus, we presume that the stimulation protocols did not contribute much to the prevalence of pregnancy loss, while the maternal age and ovarian reserve may be independent factors for predicting the risk of pregnancy loss.

After matching, the distribution of endometrial preparation regimen and embryo stages showed a slight difference between the two treatments. This was not unexpected in such large sample research. As in the Cochrane review including 18 RCTs with 3815 participating women, three regimens for FET were compared and no evidence supported the use of one cycle regimen over another [15]. The influence of embryo stages was investigated in the subgroup analysis, and both cleavage embryos and blastocysts showed the same trend between two treatments. Therefore, we presume that the two possible confounders did not contribute much to the pregnancy outcomes.

Our study has its strengths. Firstly, this trial is a large sample based on the population with a positive hCG test. The total of pregnancy outcomes during the gestation provided us with a more complete picture about the topic. Secondly, both multivariable logistic regression and propensity score matching were used to correct the baseline differences and strengthen the power to answer the research question. The results of the two statistical methods enhanced the same tendency.

Several weaknesses of the data set should not be neglected. Firstly, a common problem in retrospective studies is unmeasured confounders. Although maternal age, BMI, previous IVF attempts and oocyte yields were included and balanced in the models, it was not possible to estimate the effect of unmeasured confounders (such as education and socioeconomic status) on the ORs. However, since the matched data by propensity score stated similar ORs but narrower 95% CI owing to the high-similarity population, the coincident results provided reassurance that our findings were unlikely to be entirely explained by confounders. Secondly, as pregnancy loss is conditional upon becoming pregnant, we restricted our analysis to women with serum hCG positive tests, so our generalization was only for the conceived population. Thirdly, the possible adverse effects of progestins on the growing follicles or early-stage embryos should be further explored before the stage of embryo transfer.

## 5. Conclusions

The retrospective cohort study demonstrated that the incidence of pregnancy loss during the whole gestation and neonatal birthweights in FET cycles using embryos from PPOS were comparable to those from GnRH analogue protocols, which gave us a more complete picture about the safety of PPOS. This evidence indicated that the administration of exogenous progestins during the ovarian stimulation did not show an adverse effect on oocyte quality and subsequent embryo competence in IVF/ICSI cycles. Further evaluation should be focused on the long-term safety of the children born from PPOS.

## Figures and Tables

**Table 1:** The baseline characteristics of the matched data by propensity score.

Characteristics	GnRH Analogue (*n* = 1913)	PPOS (*n* = 3831)	*p* Value
Maternal age (yrs)	31.64 ± 3.44	31.67 ± 3.82	0.76
23–29	517 (27.0%)	1210 (31.6%)	
30–34	981 (51.3%)	1688 (44.1%)	
35–37	336 (17.6%)	640 (16.7%)	
38–42	79 (4.1%)	293 (7.6%)	
Paternal age (yrs) *n* = 14,059	33.70 ± 4.65	33.67 ± 4.97	0.81
20–34	1161 (60.7%)	2351 (61.4%)	
35–44	615 (32.1%)	1132 (29.5%)	
45–55	137 (7.2%)	348 (9.1%)	
BMI (kg/m^2^)			0.81
<18.5	238 (12.4%)	493 (12.9%)	
18.5–22.9	1175 (61.4%)	2375 (62.0%)	
23–27.4	450 (23.0%)	859 (22.4%)	
>=27.5	50 (2.8%)	104 (2.7%)	
Infertility duration (yrs)			0.69
1–3	1181 (61.7%)	2344 (61.2%)	
>=4	732 (38.3%)	1487 (38.8%)	
Gravidity			0.64
0	1014 (53.0%)	2032 (53.0%)	
1	492 (25.7%)	950 (24.8%)	
>=2	407 (21.3%)	849 (22.2%)	
No. of miscarriages *n* = 12,144			0.50
0	590 (83.8%)	3064 (85.5%)	
1	92 (13.1%)	417 (11.6%)	
>=2	22 (3.1%)	101 (2.8%)	
No. of induced abortions *n* = 12,144			0.80
0	554 (78.7%)	2815 (78.6%)	
1–2	139 (19.7%)	698 (19.5%)	
>=3	11 (1.6%)	69 (1.90%)	
Parity			0.39
0	1775 (92.8%)	3530 (92.1%)	
>=1	138 (7.2%)	301 (7.9%)	
Previous IVF attempts			0.08
0	1445 (75.6%)	2918 (76.2%)	
1–2	300 (15.7%)	530 (13.8%)	
>=3	168 (8.8%)	383 (10.0%)	
Infertility indications			0.37
Tubal	827 (43.2%)	1670 (43.6%)	
Male	285 (14.9%)	556 (14.5%)	
Endometriosis	93 (4.9%)	171 (4.5%)	
Dysfunctional ovulation	72 (3.8%)	160 (4.2%)	
Uterine	84 (4.4%)	216 (5.6%)	
Unknown	171 (8.9%)	305 (8.0%)	
Combined	381 (19.9%)	753 (19.7%)	
Basic FSH value (mIU/mL)	5.55 ± 1.57	5.56 ± 1.54	0.84
Oocyte yields			0.40
1–5	303 (15.8%)	646 (16.9%)	
6–15	1140 (59.6%)	2296 (59.9%)	
16–35	470 (24.6%)	889 (23.2%)	
Fertilization methods			0.12
IVF	1186 (62.0%)	2454 (64.1%)	
ICSI	482 (25.2%)	953 (24.9%)	
IVF + ICSI	245 (12.8%)	424 (11.1%)	
Endometrium preparation			<0.01
Natural cycle	590 (30.8%)	978 (25.5%)	
Mild stimulation	918 (48.0%)	1823 (47.6%)	
HRT	405 (21.2%)	1030 (26.9%)	
Endometrium thickness (mm) *n* = 14,231			0.37
<8 mm	125 (6.5%)	227 (5.9%)	
>=8 mm	1788 (93.5%)	3604 (94.1%)	
Embryo stage *n* = 3739			0.01
Cleavage	550 (81.7%)	2628 (85.7%)	
Blastocyst	123 (18.3%)	438 (14.3%)	
Embryos transferred			0.71
1	245 (12.8%)	504 (13.2%)	
2	1668 (87.2%)	3327 (86.8%)	

Data is shown as *n* (%).

**Table 2:** Pregnancy outcomes of positive hCG women between GnRH analogue and PPOS groups using the matched data.

	GnRH Analogue (*n* = 1913)	PPOS (*n* = 3831)	OR (95%CI)	*p* Value
**Pregnancy loss**	352 (18.4%)	734 (19.2%)	1.02 (0.97, 1.06)	0.49
Biochemical pregnancy loss	44 (2.3%)	148 (3.9%)		
Ectopic pregnancy	34 (1.8%)	115 (3.0%)		
Early miscarriage (6–11 weeks)	227 (11.9%)	333 (8.7%)		
Late miscarriage (12–24 weeks)	46 (2.4%)	134 (3.5%)		
Stillbirth (≥24 weeks)	1 (0.1%)	4 (0.1%)		
**Live birth**	1561 (81.6%)	3097 (80.8%)	0.97 (0.88, 1.06)	0.49
Gestational weeks at delivery (weeks) *n* = 4204				0.50
<=32	28 (2.8%)	89 (3.0%)		
33–36	180 (14.9%)	450 (15.0%)		
>=37	1000 (82.8%)	2457 (82.0%)		
Birth weight (g) *n* = 4617				
Single *n* (%)	1183 (76.6%)	2262 (73.5%)		
Newborn weight (g)	3346.0± 515.5	3337.0 ± 494.4		0.62
Twins	360 (23.4%)	812 (26.5%)		
Newborn weight (g)	2533.2 ± 424.2	2496.8 ± 429.2		0.18
Sex of neonates *n* = 4424				0.33
Male	682 (52.9%)	1606 (51.2%)		
Female	608 (47.1%)	1528 (48.8%)		
Mode of delivery *n* = 4593				0.15
Vaginal	318 (20.5%)	681 (22.4%)		
Cesarean section	1232 (79.5%)	2362 (77.6%)		
Low birthweight (<2500 g)	196 (12.6%)	415 (13.5%)	1.07 (0.89, 1.29)	0.46
High birthweight (>4000 g)	106 (6.9%)	176 (5.7%)	0.82 (0.64, 1.06)	0.12
Neonatal events	85 (5.5%)	155 (5.0%)	0.92 (0.70, 1.20)	0.53
Congenital anomalies	29 (1.9%)	79 (2.6%)	1.38 (0.90, 2.12)	0.14

## Data Availability

This study is according to the MDPI Research Data Policies and the datasets are available in the Department of Assisted Reproduction in Shanghai Ninth People’s Hospital.

## Supplementary Materials

The following supporting information can be downloaded at: https://www.mdpi.com/article/10.3390/jcm11206151/s1. Figure S1: The density of propensity score before and after the match; Table S1: The demographic and clinical characteristics of the original population are shown in Table S1; Table S2: The pregnancy loss and birthweights of FET cycles using embryos from PPOS and GnRH analogue using the original unmatched data (Supplemental Table S1); Table S3: The birthweights of FET cycles excluding the cases with pregnancy complications; Table S4: Pregnancy outcome of positive HCG women between GnRH analogue and PPOS groups stratified by embryo stage.
