# Supplementary material for: Comparable Pregnancy Loss and Neonatal Birthweights in Frozen Embryo Transfer Cycles Using Vitrified Embryos from Progestin-Primed Ovarian Stimulation and GnRH Analogue Protocols: A Retrospective Cohort Study
Source: J Clin Med. 2022 Oct 19;11(20):6151. doi: 10.3390/jcm11206151 (PMC9604578; doi:10.3390/jcm11206151)

Supplemental tables Pregnancy outcome of positive HCG woman (the original data)

Supplemental Table S1. The baseline characteristics of the original data.

**Characteristics**	**GnRH analogue (n=2532)**	**PPOS (n=11810)**	***P* value**
Maternal age (yrs)	31.63±3.53	31.47±3.77	0.05
23-29	806(31.8%)	4455(37.7%)	
30-34	1264(49.9%)	5117(43.3%)	
35-37	395(15.6%)	1654(14.0%)	
38-42	67(2.6%)	584(4.9%)	
Paternal age (yrs) n=14059	33.69±4.75	33.34±4.88	0.001
20-34	1640(65.3%)	8035(68.6%)	
35-44	824(32.8%)	3446(29.4%)	
45-55	49(1.9%)	234(2.0%)	
BMI (kg/m^2)			0.01
<18.5	301(11.9%)	1328(11.2%)	
18.5-22.9	1542(60.9%)	7141(60.5%)	
23-27.4	616(24.3%)	2833(24.0%)	
>=27.5	73(2.9%)	508(4.3%)	
Infertility duration (yrs)			0.01
1-3	1563(61.7%)	7755(65.7%)	
>=4	969(38.3%)	4055(34.3%)	
Gravidity			0.56
0	1357(53.6%)	6445(54.6%)	
1	642(25.4%)	2880(24.4%)	
>=2	533(21.1%)	2485(21.0%)	
No of miscarriages n=12144			0.75
0	754(84.4%)	9604(85.4%)	
1	113(12.7%)	1342(11.9%)	
>=2	26(2.9%)	305(2.7%)	
No of induced abortions n=12144			0.274
0	689(77.2%)	8909(79.2%)	
1-2	188(21.1%)	2175(19.3%)	
≥3	16(1.8%)	167(1.5%)	
Parity			0.69
0	2341(92.5%)	10946(92.7%)	
≥1	191(7.5%)	864(7.3%)	
Previous IVF attempts			<0.001
0	1909(75.4%)	9696(82.1%)	
1-2	402(15.9%)	1358(11.5%)	
≥3	221(8.7%)	756(6.4%)	
Infertility indications			<0.001
Tubal	1096(43.3%)	4209(35.6%)	
Male	376(14.8%)	1480(12.5%)	
Endometriosis	123(4.8%)	467(4.0%)	

Dysfunctional ovulation	108(4.3%)	717(6.1%)	
Uterine	106(4.2%)	704(6.0%)	
Unknown	243(9.6%)	1108(9.4%)	
Combined	480(19.0%)	3125(26.5%)	
Basic FSH value(mIU/ml)	5.52±1.54	5.68±1.63	<0.001
Oocyte yields			0.005
1-5	369(14.6%)	1622(13.7%)	
6-15	1482(58.5%)	6631(56.1%)	
16-35	681(26.9%)	3557(30.1%)	
Fertilization methods			0.226
IVF	1610(63.6%)	7279(61.6%)	
ICSI	611(24.1%)	3023(25.6%)	
IVF+ICSI	311(12.3%)	1508(12.8%)	
Endometrium preparation			<0.001
Natural cycle	725(28.6%)	2687(22.8%)	
Mild stimulation	1183(46.7%)	5739(48.6%)	
HRT	624(24.6%)	3384(28.7%)	
Endometrium thickness (mm) n=14231			0.054
<8mm	167(6.6%)	909(7.8%)	
≥8mm	2348(93.4%)	10807(92.2%)	
Embryo stage			0.001
Cleavage	721(81.3%)	8224(85.4%)	
Blastocyst	166(18.7%)	1407(14.6%)	
Embryos transferred			0.82
1	316(12.5%)	1493(12.6%)	
2	2216(87.5%)	10317(87.4%)	

Supplemental Table S2. Pregnancy outcome of positive HCG woman between GnRH analogue and PPOS groups using the original data.

	GnRH analogue (n=2532)	PPOS (n=11810)	Adjusted OR (95%CI) *	P value
**Pregnancy loss**	487(19.2%)	2231(18.9%)	0.98(0.87,1.09)	0.66
Biochemical pregnancy loss	66(2.3%)	423(3.6%)		
Ectopic pregnancy	50(2.0%)	326(2.8%)		
Early miscarriage (6-11weeks)	309(12.2%)	1027(8.7%)		
Late miscarriage (12-24 weeks)	57(2.3%)	432(3.6%)		
Stillbirth (>24weeks)	5(0.2%)	24(0.2%)		
**Live birth**	2045(80.8%)	9579(81.1%)	1.03(0.92,1.14)	0.66
**Gestational weeks at delivery n=11623**				0.089
<=32 (weeks)	38(1.9%)	258(2.7%)		
33-36 (weeks)	308(15.1%)	1456(15.2%)		

>=37 (weeks)	1699(82.1%)	7864(82.1%)		
**Birth weight (g)** n=11533				
Single n (%)	1546	6908		
Newborn weight(g)	3352.8±520.2	3338.1±504.5		0.30
Twins	473	2606		
Newborn weight(g)	2537.1±423.8	2506.2±429.4		0.15
**Mode of delivery** n=11593				0.001
Vaginal	404(19.9%)	2212(23.1%)		
Cesarean section	1628(80.1%)	7349(76.9%)		
Low birthweight (<2500g)	94(4.6%)	458(4.8%)	1.05(0.83,1.31)	0.70
High birthweight (>4000g)	343(16.8%)	1387(14.5%)	0.83(0.73,0.94)	0.005
Neonatal events	88(4.2%)	352(3.7%)	0.86(0.68,1.10)	0.23
Congenital anomalies	35(1.7%)	257(2.6%)	1.53(1.07,2.19)	0.019

* Adjusted by maternal age, paternal age, BMI, infertile duration, IVF failures and the oocyte yields.

Supplemental Table S3. The birthweights of FET cycles in matched cases without pregnancy complications

	GnRH analogue (n=1846)	PPOS (n=3607)	OR (95%CI)	P value
**Live birth**	1495(81.0%)	2874(79.7%)	0.95(0.86,1.04)	0.25
**Gestational weeks at delivery (weeks) n=4369**				0.009
<=32	27(1.8%)	73(2.5%)		
33-36	167(11.2%)	383(13.3%)		
>=37	1301(87.0%)	2317(84.1%)		
**Birth weight (g)** n=4369				
Single n (%)	1144	2134		
Newborn weight(g)	3349±515.8	3348.3±479.7		0.93
Twins	351	740		
Newborn weight(g)	2536.2±424.7	2518.9±417.2		0.53
Low birthweight (<2500g) n=4329	186(12.5%)	349(12.2%)	0.97(0.80,1.17)	0.75
High birthweight (>4000g) n=4329	104(7.0%)	165(5.8%)	0.81(0.63,1.05)	0.11
Neonatal events n=4329	68(4.6%)	99(3.5%)	0.75(0.54,1.02)	0.068
Congenital anomalies n=4329	24(1.6%)	68(2.4%)	1.48(0.93,2.37)	0.10

Supplemental Table S4. Pregnancy outcome of positive HCG woman between GnRH analogue and PPOS in matched cases stratified by embryo stage

	GnRH analogue (n=1913)	PPOS (n=3831)	OR (95%CI)	P value
**Cleavage embryo transfer**				
Live birth	1461(81.6%)	2740(80.8%)	1.01(0.97,1.05)	0.64
Gestational weeks at delivery n=3747				0.75
<=32 weeks	27(2.4%)	76(2.9%)		
33-36 weeks	167(15.1%)	397(15.0%)		
>=37 weeks	914(82.5%)	2166(82.1%)		
Birth weight (g) n=4165				
Single n (%)	1092	1968		
Newborn weight(g)	3342.9±515.5	3331.4±487.2		0.54
Twins	351	754		
Newborn weight(g)	2430.2±423.1	2504.6±421.5		0.35
**Blastocyst transfer**				
Live birth	100(81.3%)	357(81.5%)	1.0(0.90,1.12)	0.96
Gestational weeks at delivery n=457				0.34
<=32 weeks	1(1.0%)	13(3.6%)		
33-36 weeks	13(13.0%)	53(14.8%)		
>=37 weeks	86(86.0%)	291(81.5%)		
Birth weight (g) n=452				
Single n (%)	91	294		
Newborn weight(g)	3383.7±517.0	3374.0±540.0		0.88
Twins	9	58		
Newborn weight(g)	2648.3±477.2	2394.6±512.7		0.17

Figure S1: The density of propensity score before and after the match.

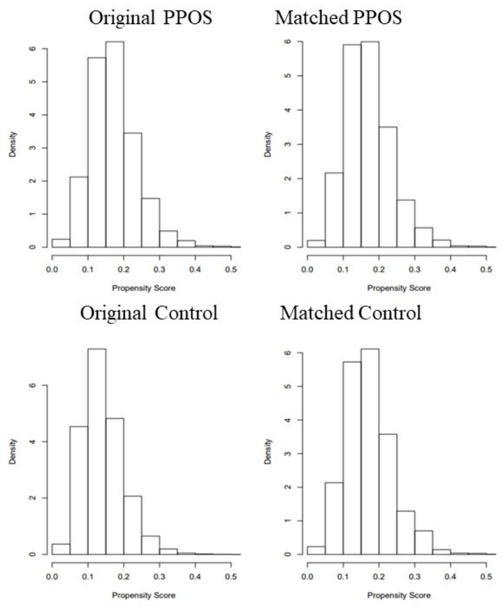

Supplement: Supplementary file 1 [file jcm-11-06151-s001.zip › jcm-1877169-Supplementary.pdf]
